# Fluorescently Tagged Poly(methyl methacrylate)s

**DOI:** 10.3390/molecules29245940

**Published:** 2024-12-16

**Authors:** Fabia Grisi, Rubina Troiano, Donatella Fiore, Patrizia Gazzerro, Mariateresa Lettieri, Vincenzo Venditto, Stefania Pragliola

**Affiliations:** 1 Dipartimento di Chimica e Biologia, and INSTM Research Unit, Università di Salerno, Via Giovanni Paolo II 132, I-84084 Fisciano, SA, Italy; fgrisi@unisa.it (F.G.); rutroiano@unisa.it (R.T.); vvenditto@unisa.it (V.V.); 2 Dipartimento di Farmacia, Università di Salerno, Via Giovanni Paolo II, 132, I-84084 Fisciano, SA, Italy; dfiore@unisa.it (D.F.); pgazzerro@unisa.it (P.G.); 3 CNR-SPIN, c/o Dipartimento di Fisica, Università di Salerno, Via Giovanni Paolo II, 132, I-84084 Fisciano, SA, Italy; mariateresa.lettieri@cnr.it

**Keywords:** poly(methyl metacrylate), carbazole, coumarin, fluorescent tagging, plastic recycling

## Abstract

Plastic pollution is a global problem affecting the environment and, consequently, people’s well-being. Careful and timely end-of-life plastic recycling is certainly a way, albeit a partial one, to remedy the problem. The immediate identification and selection of the different types of plastic materials in the recycling process certainly facilitate its recovery and reuse, allowing the damage caused by plastic emission into the environment to be limited. Recently, new technologies for automatic sorting of plastics based upon fluorescent tagging have been considered. This article reports the synthesis and characterization of fluorescent copolymers of poly(methyl methacrylate) (PMMA) that could be potentially used as fluorescent markers of commercial PMMA. Poly(methylmetacrylate-*co*-2-(9-carbazolyl)ethyl methacrylate) (P(MMA-*co*-CEMA)) and poly(methylmetacrylate-*co*-7-methacryloyloxycoumarin) (P(MMA-*co*-MAOC)) samples containing a small number of fluorescent units (<4%) were synthesized by free-radical polymerization. All copolymer samples show chemico-physical properties like those of pure PMMA and produce fluorescence emission under 290 nm wavelength excitation. P(MMA-*co*-CEMA)s and P(MMA-*co*-MAOC)s were also tested as fluorescent dyes for PMMA identification. The experimental results demonstrate that PMMA/P(MMA-*co*-CEMA) and PMMA/P(MMA-*co*-MAOC) blends prepared using 1% by weight of fluorescent copolymer show a homogeneous morphology completely similar to pure PMMA and are still optically active.

## 1. Introduction

**Scheme 1:**
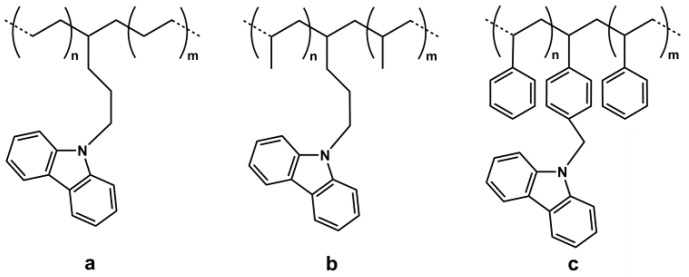
PE (**a**), PP (**b**), and PS (**c**) fluorescent copolymer structures as reported in Refs. [16,17].

Although it is unanimously acknowledged that plastics play a key part in improving the quality of life of people, their impact on the natural environment has been catastrophic. The continuing rise of non-biodegradable plastic products placed on the market and, above all, their indiscriminate and reckless end-of-life disposal lead to devastating consequences, the most glaring of which is probably the well-known Pacific waste vortex [1]. Furthermore, plastic waste, inconsiderately disposed of in the environment, slowly degrades due to biological, chemical, and climatic factors, converting into micro- and nanoplastics (MPs and NPs) that are extremely harmful to both terrestrial and aquatic ecosystems [2,3,4,5]. MPs and NPs can cross the food chain via biomagnification and bioaccumulation within living organisms, up to humans. As a matter of fact, plastic pollution is currently one of the most pressing environmental issues facing humanity as a whole. Plastic pollution can be greatly reduced by recycling plastic. Recycled plastic waste can be used to create new products with a consequent significant drop in the volume of plastic waste in the environment. In recent years, increasingly sophisticated technologies leading to improvements in plastic garbage collection, sorting, and recycling have been developed [6]. Nowadays, selecting systems based on the microstructural and chemico-physical characteristics of the polymers are exploited in mechanical plastic recycling processes. Infrared and near-infrared spectroscopy, which enables us to identify the presence of the chemical functional groups of a given substance, is widely applied for this purpose [7,8,9,10]. However, due to biological, chemical, and environmental factors, plastic waste can undergo slow degradation over time, and therefore, spectroscopic methods can become ineffective for their identification. Fluorescent tagging is another method that can be adopted for the identification of different plastic materials. Distinctive fluorescent labels can be applied to different polymers, and fluorescent dye can either be applied as a coating on plastic surfaces or blended with polymers during the compounding process [11,12,13,14,15]. Although fluorescent labeling is a very cheap and particularly sensitive technique allowing effective detection even in the presence of very low concentrations of the tracer, it has some disadvantages that could make it ineffective. Fluorescent labels can easily be accidentally removed while using a plastic item. Fluorescent coatings cannot be applied to polyolefin materials due to their poor adhesiveness. Additionally, it is advised to select suitable tracers that do not decompose during plastic separation procedures, which typically entail high temperatures, and that remain stable over the course of a plastic item’s life. In recent articles, some of us have reported the synthesis and the characterization of new fluorescent copolymers of common plastic materials such as polypropylene (PP), polyethylene (PE), and polystyrene (PS) (see Scheme 1) [16,17]. Such materials contain a very low number of fluorescent units (less than 4%) and exhibit chemico-physical properties like those of the corresponding commercial homopolymers. Thus, they seem suitable for serving as fluorescent identifiers of their homologous homopolymers. They should be especially well suited for use as fluorescent labels of plastics as it was demonstrated that the addition of minimal amounts of fluorescent copolymer to the corresponding homopolymer produces fluorescence-traceable homogeneous blends [16,17]. The synthesized fluorescent copolymers can be used for fluorescent tagging-based MP and NP pollutant detection in organic tissues too. It was demonstrated that MPs and NPs derived from such tagged copolymers are readily identifiable within animal tissues through confocal microscopy, leading the way for the use of labeled copolymers to inquire how the biota is affected by MP and NP pollution [17,18].

Although in the global plastic market polymethylmetacrylate (PMMA) is produced in a much lower volume compared to other common polymers such as polyolefins, PMMA market demand is constantly growing due to its unique properties like UV resistance, high transparency, lightness, and barrier properties [19,20]. All these excellent features make PMMA particularly suitable for optical and electronic applications. Compared to other common plastics, the production costs of PMMA are quite high, such that, in recent years, particular attention has been paid to the development of new technologies for recovering and reusing it. Despite this, only 10% of global PMMA production is currently recycled, while the majority of post-consumer PMMA ends up in landfills, becoming a persistent environmental pollutant [19,20,21]. In this paper, we report the synthesis and the microstructural and chemico-physical characterization of PMMA fluorescent copolymers such as poly(methylmetacrylate-*co*-2-(9-carbazolyl)ethyl methacrylate)s (P(MMA-*co*-CEMA)s) and poly(methyl metacrylate-*co*-7-methacryloyloxycoumarin)s (P(MMA-*co*-MAOC)s). The P(MMA-*co*-CEMA)s and P(MMA-*co*-MAOC)s’ optical characterization was also performed in order to investigate their possible applications as fluorescent markers of commercial PMMA, allowing their identification throughout the sorting phase of the plastic recycling process.

## 2. Results and Discussion

### 2.1. Synthesis of and Microstructural, Chemico-Physical, and Optical Characterization of P(MMA-co-CEMA)s and P(MMA-co-MAOC)s

**Scheme 2:**
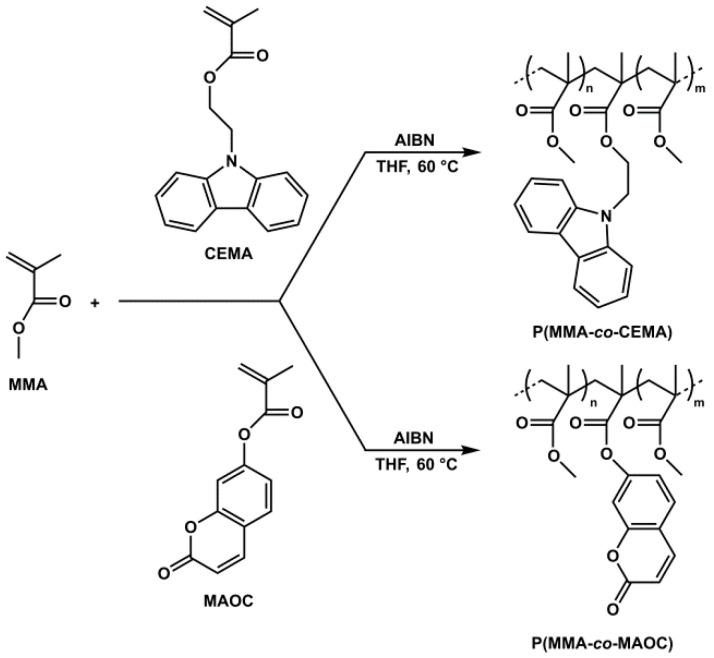
Copolymer synthesis.

Poly(methyl metacrylate-*co*-2-(9-carbazolyl)ethyl methacrylate) (P(MMA-*co*-CEMA)) and poly(methyl metacrylate-*co*-7-methacryloyloxycoumarin) (P(MMA-*co*-MAOC)) samples were prepared by free-radical polymerization using 2,2′-azobis(isobutyronitrile) (AIBN) as an initiator (see Scheme 2).

Our primary objective was to achieve fluorescent PMMA samples capable of giving homogeneous blends with PMMA, meaning they would have chemico-physical characteristics that are almost identical to those of pure PMMA. Consequently, every copolymerization experiment was conducted such that the content of fluorescent comonomer units inserted into the copolymer chains did not surpass 4%. Table 1 provides a summary of the findings.

**Figure 1:**
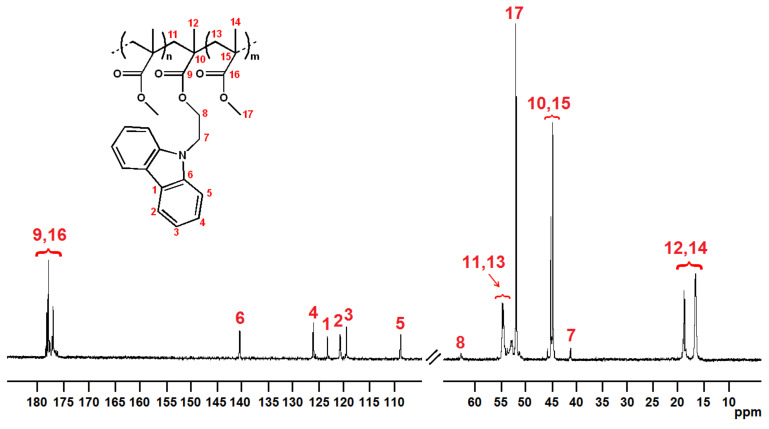
^13^C NMR spectrum of run **3** (CDCl_3_ solvent, TMS scale, 25 °C).

**Figure 2:**
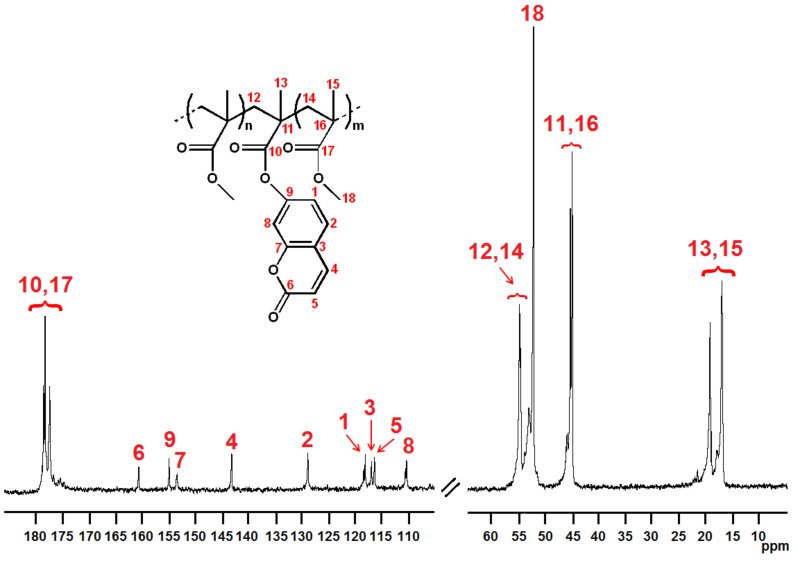
^13^C NMR spectrum of run **6** (CDCl_3_ solvent, TMS scale, 25 °C).

The copolymer microstructures were evaluated by ^13^C NMR analysis (see Figure 1 and Figure 2). The assignments were made taking into account the data already reported in the literature [22,23] and are listed in Tables S1 and S2 of the Supplementary Materials of this article. For both copolymer series, P(MMA-*co*-CEMA)s and P(MMA-*co*-MAOC)s, the fluorescent unit content was evaluated via ^13^C NMR, comparing the areas of the signals of the α methyl carbons of both MMA and fluorescent units and the ester methyl carbons of MMA units. In detail, an equation was applied as reported below:

%F (fluorescent unit (CEMA or MAOC) fraction) = [(A_α_−A_es_)/A_α_] × 100

where

F is the molar fraction of the fluorescent unit (CEMA or MAOC);
Aα is the area of the signals of the α methyl carbons of both MMA and fluorescent (CEMA or MAOC) units;
Aes is the area of the signals of the ester methyl carbons of MMA units.

The molar compositions of the copolymers for both the series completely reflect that of monomers in the feed (see Table 1), proving that each monomer (MMA, CEMA, and MAOC) present similar reactivity, at least in the used experimental conditions. All synthesized samples are random copolymers; however, although it was not possible to experimentally determine the distribution of the fluorescent units in the copolymer chains through NMR analysis, considering that for each copolymerization test a much lower amount of fluorescent monomer than MMA was used as well as a similar reactivity value for each monomer, it is reasonable to assume that the fluorescent units are homogeneously distributed throughout the polymer chains. This means that the polymer chains likely consist of long sequences of MMA units interspersed with mostly isolated fluorescent units.

**Figure 3:**
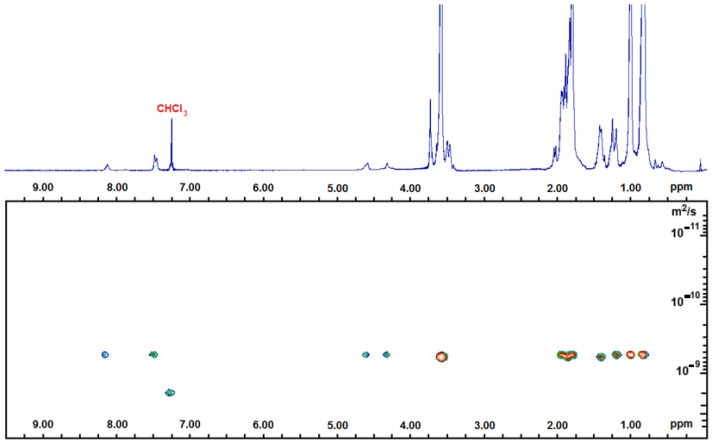
The 600 MHz 2D DOSY NMR spectrum of run **3** (CDCl_3_ solvent, TMS scale, 25 °C).

**Figure 4:**
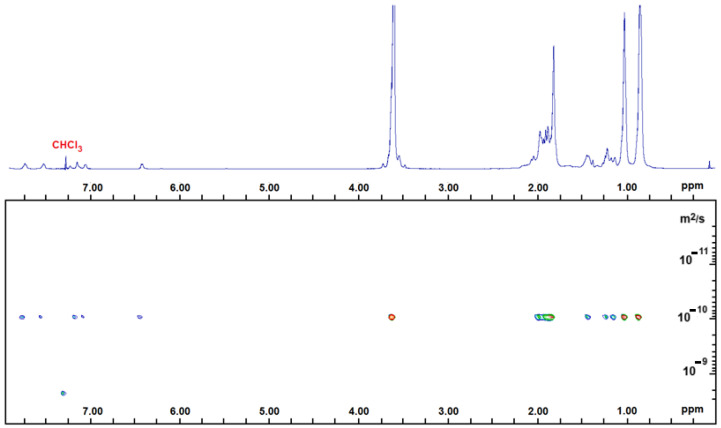
The 600 MHz 2D DOSY NMR spectrum of run **6** (CDCl_3_ solvent, TMS scale, 25 °C).

The effectiveness of the copolymerization reaction for both copolymer series was verified by DOSY NMR analysis. As an example, the DOSY maps with the ^1^H NMR spectra projected on top of run **3** (P(MMA-*co*-CEMA)) and run **6** (P(MMA-*co*-MAOC)), showing that both ^1^H NMR signals of the fluorescent comonomer isolated units and the MMA homosequences have the same diffusion coefficient, are reported in Figure 3 and Figure 4. The molecular weights and dispersity of the copolymer samples were determined by SEC analysis (Figures S1–S6 in Supplementary Materials). All exhibit monomodal molecular-weight distribution curves, indicating their homogeneity. The dispersity values also match those expected for polymer samples produced by free-radical polymerization.

**Figure 5:**
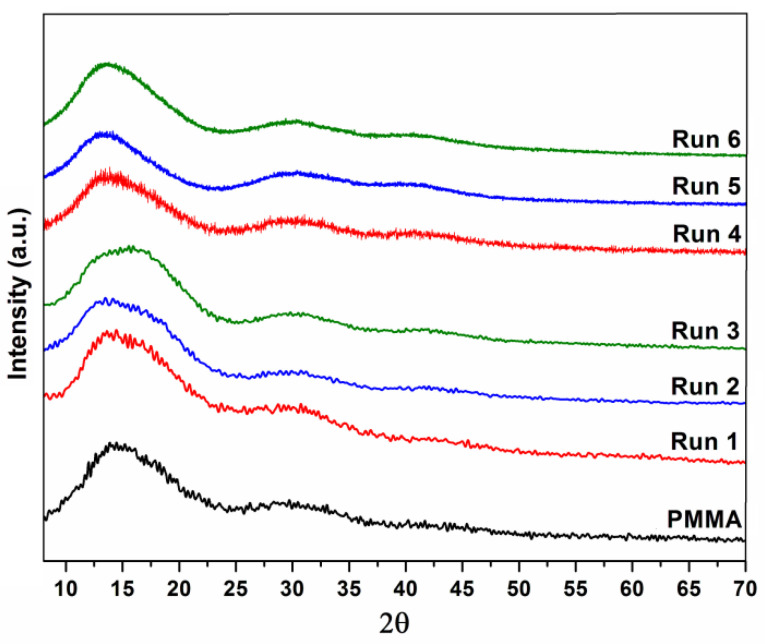
X-ray diffraction patterns of runs **1**–**6** from Table 1 and amorphous PMMA sample.

The chemico-physical features of all fluorescent copolymers were compared with pure PMMA. In Figure 5, the X-ray diffraction patterns of (P(MMA-*co*-CEMA) and P(MMA-*co*-MAOC) and pure amorphous PMMA samples are depicted. Similarly to the PMMA spectrum, both P(MMA-*co*-CEMA)_s_ and P(MMA-*co*-MAOC)_s_ show spectra having the typical broad diffraction peaks at 2θ = 14° due to intermolecular correlations and at 30° and 42° attributable to intramolecular ones [24].

**Figure 6:**
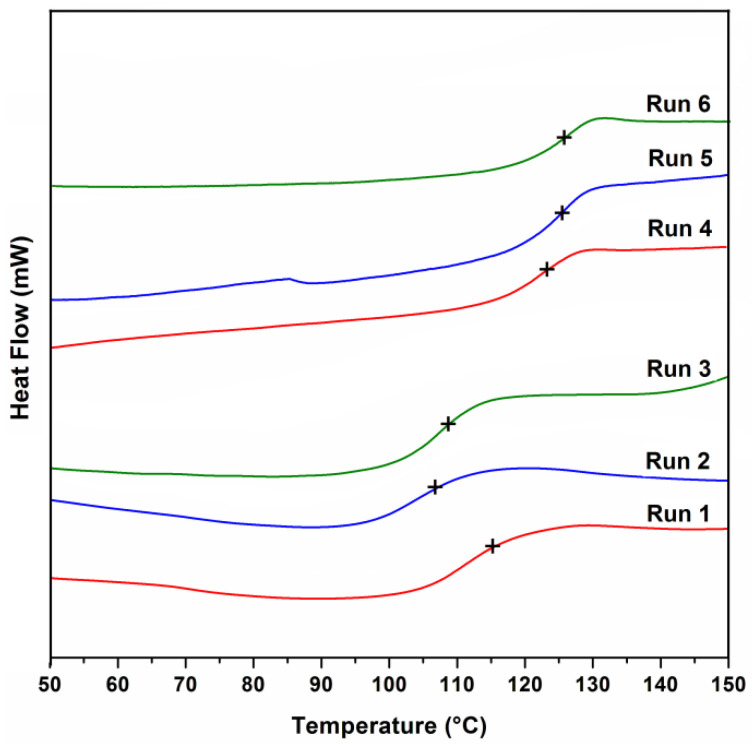
DSC curves (second heating run) of runs **1**–**6** from Table 1.

Regarding the thermal analysis, both P(MMA-*co*-CEMA)_s_ and P(MMA-*co*-MAOC)_s_ exhibit thermogravimetric curves comparable with that of pure PMMA [25,26]. As reported for PMMA, the thermogravimetric curve of each copolymer sample (see Figures S7–S12 in Supplementary Materials) presents three inflections at approximately 115, 275, and 370 °C due to cleavages of head-to-head linkage, cleavages at the unsaturated ends due to termination by disproportionation, and random cleavages within the polymer chain, respectively. The DSC analysis confirms the amorphous nature of both P(MMA-*co*-CEMA)_s_ and P(MMA-*co*-MAOC)_s_, and as shown in Figure 6, only a single glass transition phenomenon is detected in any copolymer thermogram. The fluorescent copolymer T_g_ values are always comparable to those of pure PMMA with comparable molecular weight [25,26].

**Figure 7:**
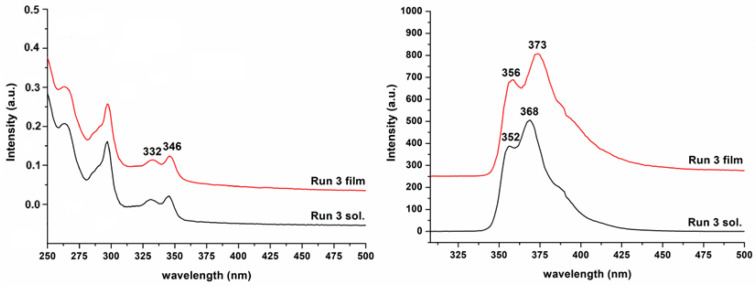
UV-vis absorption and emission spectra (Λ_ecc_ = 290 nm) of run **3** in solution and solid state.

**Figure 8:**
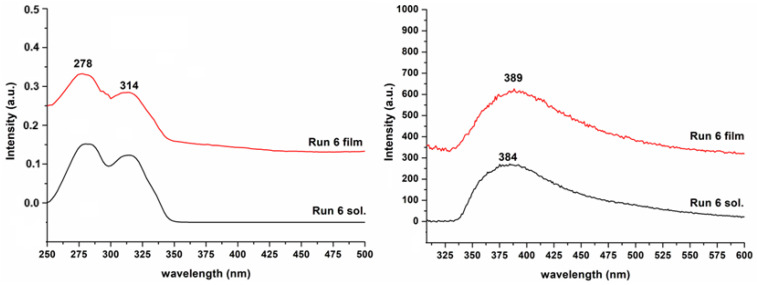
UV-vis absorption and emission spectra (Λ_ecc_ = 290 nm) of run **6** in solution and solid state.

UV-vis absorption and emission spectra of both P(MMA-*co*-CEMA)_s_ and P(MMA-*co*-MAOC)_s_ in solution and the solid state were recorded, and as an example, the collected spectra of runs **3** and **6** are reported in Figure 7 and Figure 8. The P(MMA-co-CEMA) UV-vis absorption spectra present two peculiar bands at 332 and 346 nm deriving from π-π* absorption of the carbazole group [27,28,29]. Rather, the P(MMA-co-MAOC) spectra have two characteristic bands at 278 and 314 nm, which are caused by π–π* transitions from the electrons of the pyrone nucleus and the benzene nucleus, respectively [30,31]. Both solution and film emission spectra of P(MMA-*co*-CEMA)_s_, recorded under an excitation wavelength of 290 nm, reveal in the high-energy region two bands assigned to carbazole group emission [27,28,29,31]. With a broad band in the 350–500 nm range, the solution and film emission spectra of P(MMA-co-MAOC)s have a spectral profile that is entirely identical to that of the monomer MAOC. Comparing the emission spectra of both P(MMA-*co*-CEMA) and P(MMA-*co*-MAOC) copolymers in solution and in the solid state, only a very slight bathochromic shift in the emission bands in the solid state, with respect to the solution one, can be detected.

### 2.2. Fluorescence Property Study of P(MMA-co-CEMA)s and P(MMA-co-MAOC)s

**Figure 9:**
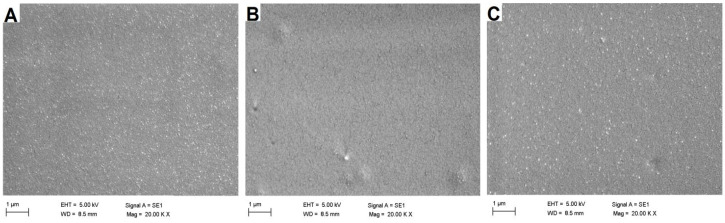
SEM micrographs of (**A**) pure PMMA, (**B**) PMMA/P(MMA-co-CEMA) blend, and (**C**) PMMA/P(MMA-co-MAOC) blend films.

Since both P(MMA-*co*-CEMA)s and P(MMA-*co*-MAOC)s exhibit strikingly similar chemico-physical properties to pure PMMA and are also optically active, they were tested as fluorescent labels for environmental applications. In order to investigate their potential uses as fluorescent markers of commercial PMMA, allowing its identification throughout the sorting phase of the plastic recycling process, films of polymer blends containing pure PMMA and 1% by weight of P(MMA-*co*-CEMA) (run **3**) or P(MMA-*co*-MAOC) (run **6**) were prepared by solution-casting. Morphological examination by means of SEM was performed on the fractured surfaces of the PMMA/P(MMA-*co*-CEMA) and PMMA/P(MMA-*co*-MAOC) blend films and, for comparison, of a pure PMMA film sample. The SEM micrographs are reported in Figure 9. Both the polymer blends have a homogeneous morphology that is completely similar to that of pure PMMA, and as expected, this indicates a fine dispersion and homogeneous incorporation of the fluorescent copolymers into PMMA.

**Figure 10:**
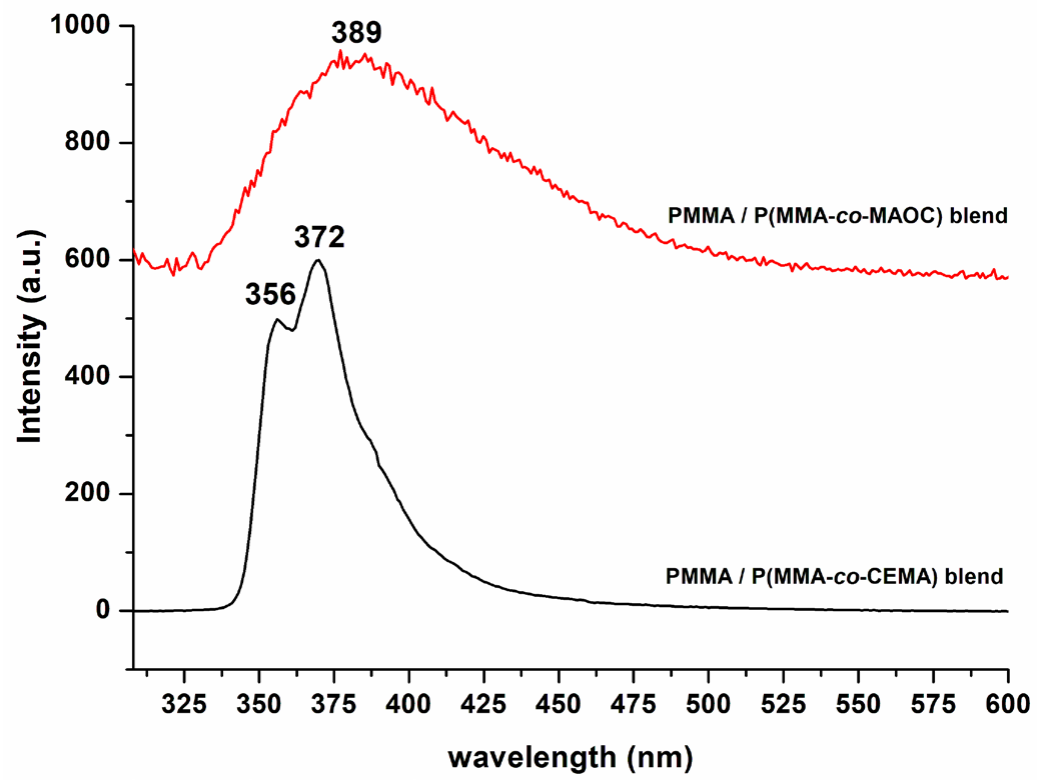
Emission spectra of the PMMA/P(MMA-*co*-CEMA) and PMMA/P(MMA-*co*-MAOC) blends containing 1% by weight of P(MMA-*co*-CEMA) and P(MMA-*co*-MAOC), respectively (Λ_ecc_ = 290 nm).

The emission spectra of PMMA/P(MMA-*co*-CEMA) and PMMA/P(MMA-*co*-MAOC) blend films, recorded at room temperature using an excitation wavelength of 290 nm, are shown in Figure 10. Both show a shape that is completely similar to that of the respective fluorescent copolymers. Like P(MMA-*co*-CEMA), the PMMA/P(MMA-*co*-CEMA) blend spectrum shows bands at 356 and 373 nm attributable to the emission of the carbazole units. As regards the PMMA/P(MMA-*co*-MAOC) blend spectrum, the band at 389 nm assigned to the emission of coumarin groups is detectable.

**Figure 11:**
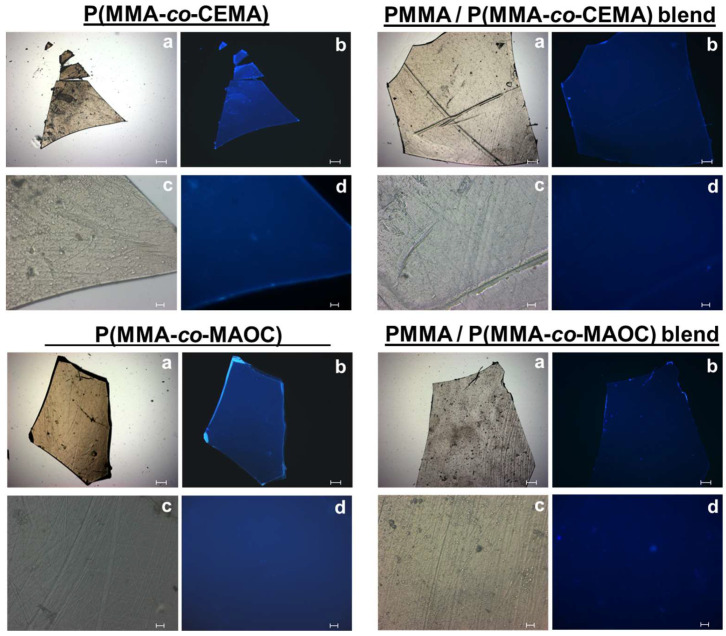
Representative images of P(MMA-*co*-CEMA), PMMA/P(MMA-*co*-CEMA) blend, P(MMA-*co*-MAOC), and PMMA/P(MMA-*co*-MAOC) blend fluorescence properties. In (**a**) and (**c**), brightfield images are shown. Panels (**b**) and (**d**) show blue fluorescence (analyzed with DAPI filters: 340–360 nm excitation range and 440–460 nm emission range). Scale bars in (**a**) and (**b**) = 1000 µm; scale bars in (**c**) and (**d**) = 200 µm.

To further prove the high potential of the application of our fluorescent copolymers for plastic discrimination, PMMA/P(MMA-*co*-CEMA) and PMMA/P(MMA-*co*-MAOC) blend thin-film samples and, for comparison, P(MMA-*co*-CEMA) and P(MMA-*co*-MAOC) ones were also observed by using fluorescence microscopy analysis. Figure 11 shows the fluorescence micrographs of film sample fragments of both blends and, for comparison, those of P(MMA-*co*-CEMA) and P(MMA-*co*-MAOC) ones. Although the blend fluorescent signals are slightly weaker than P(MMA-*co*-CEMA) and P(MMA-*co*-MAOC) ones, both PMMA/P(MMA-*co*-CEMA) and PMMA/P(MMA-*co*-MAOC) blends are clearly recognizable as fluorescence in blue. As expected, the fluorescence micrograph of the pure PMMA film sample (see Figure S13 in Supplementary Materials) appears completely dark. The experimental results, therefore, seem to indicate that both P(MMA-*co*-CEMA) and P(MMA-*co*-MAOC) copolymers can be considered as suitable fluorescent markers of PMMA. Indeed, the addition of just 1% by weight of fluorescent copolymer to pure PMMA leads to fluorescent homogeneous blends easily selectable from other types of plastics.

## 3. Materials and Methods

### 3.1. Materials

All the manipulations involving compounds that are sensitive to air and moisture were performed using Schlenk techniques under an inert atmosphere of nitrogen using Schlenk techniques. All glassware and vials were stored in an oven at 120 °C overnight and subjected to three vacuum–nitrogen cycles prior to use. All reagents and solvents were obtained from Sigma-Aldrich s.r.l. (Milano, Italy). Tetrahydrofuran was dried before use by distillation over lithium aluminum hydride. The initiator, azobisisobutyronitrile (AIBN) (purity 98%), was further purified by recrystallization in methanol before use.

### 3.2. Measurements

The NMR spectra of all monomers and polymers were registered on an ASCEND 600 spectrometer (Bruker, Ettlingen, Germany) (^1^H, 600 MHz; ^13^C, 150 MHz) operating at 298 K. NMR tubes of samples were prepared by dissolving 5 mg of the selected product in 0.5 mL of deuterated chloroform (CDCl_3_). Tetramethylsilane (TMS) was used as an internal reference for calibrating chemical shift values.

The molecular masses (M_n_ and M_w_) and dispersity (M_w_/M_n_) of all copolymer samples were determined at 25 °C by an Agilent GPC/SEC instrument (Agilent, Santa Clara, CA, USA), using THF as the mobile phase (1.0 mL/min) and narrow polystyrene standards as the reference.

Thermogravimetric analysis (TGA) measurements were performed on a TGA Q500 apparatus manufactured by TA Instruments (Waters/TA instruments, New Castle, DE, USA) in flowing N_2_ (100 cm^3^/min). Each sample was prepared by placing 5 mg of polymer into a platinum pan and heating at 10 °C/min from 20 to 800 °C.

Thermal analysis was carried out by using a TA-DSC Q20 apparatus manufactured by Waters/TA instruments (Waters, New Castle, DE, USA) in flowing N_2_. A total of 5 to 7 mg of each polymer sample was sealed into an aluminum pan and heated/cooled in a range of 0–200 °C at 10 °C/min.

Wide-angle X-ray diffraction (WAXD) measurements were carried out on the polymer powder samples using a Bruker D8 Advance automatic diffractometer (Bruker, Karlsruhe, Germany), in reflection, by Ni-filtered Cu-Ka average radiation (1.5418 Å).

UV-vis spectra of the copolymer samples were obtained using a Varian Cary 50 spectrophotometer (Agilent, Santa Clara, CA, USA), while photoluminescence spectra were recorded on a Varian Cary Eclipse spectrophotometer (Agilent, Santa Clara, CA, USA). Solid-state measurements were performed on polymer thin (100 nm) films, deposited on a quartz substrate, prepared by spin-coating from chloroform polymer solutions (1 mg/mL) in air at 500 rpm for 60 s.

The morphologies of the blend film samples were observed via scanning electron microscopy (SEM) using a LEO Evo 50 microscope (Carl Zeiss AG, Oberkochen, Germany). The cross-sectional specimens of the film samples were obtained in liquid nitrogen by brittle fractures. To improve SEM image quality, the films were sputter-coated with a very thin layer of gold. This treatment reduces the charging effects of the surface, enhancing its conductivity while preserving its morphology. The fractured surfaces were examined, collecting the SEM images at an acceleration voltage of 5 kV, a working distance of 8.5 mm, and a beam current of 30 μA.

To analyze fluorescence properties, P(MMA-*co*-CEMA), PMMA/P(MMA-*co*-CEMA) blend, P(MMA-*co*-MAOC), PMMA/P(MMA-*co*-MAOC) blend, and pure PMMA films were mounted on microscope slides and examined under an Axioplan 2 Imaging fluorescence microscope, equipped with an AxioCam HRc Camera (Carl Zeiss), using 2.5× and 10× objectives, and then analyzed through AxioVision 4.8 software. For each sample, at least six different fields were examined. Fluorescence was analyzed using DAPI filters with a 340–360 nm excitation range and a 440–460 nm emission range (blue fluorescence).

### 3.3. Synthesis of the Monomer 2-(9-carbazolyl)ethyl Methacrylate (CEMA)

The monomer, 2-(9-carbazolyl)ethyl methacrylate, was synthesized by using a two-step procedure as already reported in Ref. [32]. Carbazole (10.0 g, 0.0598 mol) and ethylene carbonate (7.90 g, 0.0897 mol) were reacted in 200 mL of refluxing dry dimethylformamide (DMF) for 4 h in the presence of a small amount of sodium hydroxide. After cooling to room temperature, the mixture was extracted with benzene. The benzene solution was washed with water five times and then dried over anhydrous MgSO_4_ for 2 h and filtered. The crude product (9-(2-hydroxyethyl)carbazole) was obtained by removing the solvent (benzene) under vacuum. It was purified by crystallization using a chloroform/hexane mixture (10.1 g). Yield: 80%. Following this, to a 200 mL dichloromethane (CH_2_Cl_2_) solution of 9-(2-hydroxyethyl) carbazole (4.10 g, 0.0194 mol) and triethylamine (2.49 g, 0.0246 mol), methacryloyl chloride (3.04 g, 0.0291 mol) was added at 0 °C. The mixture was stirred for 3 h at room temperature and then poured into water. The product was extracted three times with CH_2_Cl_2_. The organic layer was dried over anhydrous MgSO_4_ and filtered, and the solvent was removed by evaporation to obtain the crude product. Recrystallization from methanol solution produced pure 2-(9-carbazolyl)ethyl methacrylate as white crystals (4.33 g). Yield: 80%. T_m_: 82.1 °C; ^1^H NMR (CDCl_3_) δ: 1.82 (s, 3H, methyl), 4.56 (t, 2H, methylene), 4.63 (t, 2H, methylene), 5.48 (m, 1H, vinyl), 5.93 (s, 1H, vinyl), 7.23–7.45 (m, 6H, aromatic), 8.10 (d, 2H, aromatic); ^13^C NMR (CDCl_3_) δ: 168.92, 140.37, 138.20, 126.34, 125.75, 123.01, 120.37, 119.24, 108.58, 62.46, 41.60, 18.20.

### 3.4. Synthesis of the Monomer 7-Methacryloyloxycoumarin (MAOC)

The monomer, 7-methacryloyloxycoumarin, was synthesized by slightly modifying the procedure already reported in Ref. [33]. To a 200 mL dry dimethylformamide solution of 7-hydroxycoumarin (27.6 g, 0.170 mol) and triethylamine (20.2 g, 0.200 mol), methacryloyl chloride (20.9 g, 0.200 mol) was added at 0 °C. The mixture was refluxed for 4 h and then cooled at room temperature and poured into water and ice. The crude product was filtered, washed with water several times, and dried. Recrystallization from ethanol solution produced pure 7-methacryloyloxycoumarin as pale-yellow crystals (31.3 g). Yield: 80%. T_m_: 140.3 °C; ^1^H NMR (CDCl_3_) δ: 2.07 (s, 3H, methyl), 5.82 (s, 1H, vinyl), 6.38 (s, 1H, =CHCOO of coumarin ring), 6.41 (s, 1H, vinyl), 7.06–7.72 (4H, coumarin ring); ^13^C NMR (CDCl_3_) δ: 165.10, 160.36, 154.72, 153.50, 142.85, 135.32, 128.51, 128.25, 118.47, 116.61, 116.05, 110.50, 18.30.

### 3.5. Methyl Methacrylate (MMA)-2-(9-carbazolyl)ethyl Methacrylate (CEMA) Copolymerizations

In a 50 mL round-bottom flask equipped with a magnetic stir bar, 2-(9-carbazolyl)ethyl methacrylate (run 1: 0.280 mmol, 0.0780 mg; run 2: 0.56 mmol, 0.156 g; run 3: 1.12 mmol, 0.312 g) and methyl methacrylate (28 mmol (3.0 mL)) were dissolved in 10 mL of dry THF. Azobisisobutyronitrile (AIBN) (0.0343 mmol, 0.0564 g) was added to the reaction mixture, and the flask was warmed at 60 °C for 4 h. The mixture was then poured into a large excess of ethanol, and the white precipitate was recovered by filtration. After washing with fresh ethanol, the polymer was dried at room temperature in vacuum.

### 3.6. Methyl Methacrylate (MMA)-7-Methacryloyloxycoumarin (MAOC) Copolymerizations

In a 50 mL round-bottom flask equipped with a magnetic stir bar, 7-methacryloyloxycoumarin (run 4: 0.280 mmol, 0.0644 g; run 5: 0.56 mmol, 0.129 g; run 6: 1.12 mmol, 0.258 g) and methyl methacrylate (28 mmol (3.0 mL)) were dissolved in 10 mL of dry THF. Azobisisobutyronitrile (AIBN) (0.0343 mmol, 0.0564 g) was added to the reaction mixture, and the flask was warmed at 60 °C for 2 h. The mixture was then poured into a large excess of ethanol, and the white precipitate was recovered by filtration. After washing with fresh ethanol, the polymer was dried at room temperature in vacuum.

### 3.7. Preparation of PMMA/P(MMA-co-CEMA) and PMMA/P(MMA-co-MAOC) Blends

PMMA/P(MMA-*co*-CEMA) and PMMA/P(MMA-*co*-MAOC) polymer blend samples were prepared by dissolving 500 mg of pure PMMA and 5 mg of P(MMA-*co*-CEMA) or P(MMA-*co*-MAOC) in 20 mL of chloroform at 50 °C under stirring. The homogeneous polymer mixture was then poured into an excess of ethanol, and the precipitate was recovered by filtration and dried under vacuum at 40 °C overnight. Polymer blend films were prepared from their chloroform solutions (5 mg/mL) using the solution-casting method.

## 4. Conclusions

Two series of fluorescent PMMA copolymers, P(MMA-*co*-CEMA)s and P(MMA-*co*-MAOC)s, were prepared via free-radical polymerization. The low number of fluorescent units contained in the polymer chains means that their chemico-physical properties are almost identical to those of pure PMMA samples of similar molecular weight and, at the same time, makes them traceable by fluorescence. In order to test whether both P(MMA-*co*-CEMA)_s_ and P(MMA-*co*-MAOC)_s_ could be suitable as fluorescent markers of PMMA, PMMA/P(MMA-*co*-CEMA) and PMMA/P(MMA-*co*-MAOC) blends containing 1 wt% fluorescent polymer were prepared. In the SEM analysis, the blend film samples showed a homogeneous morphology completely like that of pure PMMA while retaining the same optical feature of P(MMA-*co*-CEMA) or P(MMA-*co*-MAOC) film samples. Furthermore, fluorescence micrographs of film sample fragments of both blends showed that they are clearly recognizable because they fluoresce blue. Finally, it is worth noting that, due to their unique characteristics, both P(MMA-*co*-CEMA) and P(MMA-*co*-MAOC) copolymers can find applications not only for the fluorescent tagging of pure PMMA, allowing its identification in plastic recycling processes, but also to detect PMMA-derived NP or MP contaminants. Since both P(MMA-*co*-CEMA) and P(MMA-*co*-MAOC) copolymers exhibit the same chemico-physical properties as pure PMMA and are clearly identifiable through fluorescence microscopy, they could also be used as PMMA substitutes for investigating the impact of PMMA-derived microplastic pollutants in model organisms.

## Figures and Tables

**Table 1:** Polymerization results.

Run ^a^	MMA ^b^ (mmol)	CEMA ^b^ (mmol)	Yield (%)	CEMA ^c^ (%)	M_n_^d^ (10^3^Da)	Ð ^d^	T_g_^e^ (°C)
1	28	0.28	58	1	29.8	1.3	115.5
2	28	0.56	62	2	18.3	1.5	107.9
3	28	1.12	66	4	22.3	1.3	109.6
**Run ^a^**	**MMA ^b^** **(mmol)**	**MAOC ^b^** **(mmol)**	**Yield** **(%)**	**MAOC ^c^** **(%)**	**M_n_^d^** **(10^3^Da)**	**Ð ^d^**	**T_g_^e^** **(°C)**
4	28	0.28	31	1	21.4	1.8	123.4
5	28	0.56	25	2	18.4	1.4	125.2
6	28	1.12	20	4	18.5	1.5	125.5

^a^ Polymerization conditions: solvent = THF (10 mL), temperature = 60 °C, time = 4 h for runs 1, 2, and 3 and 2 h for runs 4, 5, and 6. ^b^ Comonomer concentration in the feed. ^c^ The % of fluorescent comonomer units in the copolymer chains was evaluated by ^13^C NMR. ^d^ Determined by THF size exclusion chromatography (SEC), polystyrene-standard-calibrated. ^e^ Evaluated on second DSC heating run.

## Data Availability

The data presented in this study are available on request from the corresponding author.

## Supplementary Materials

The following supporting information can be downloaded at https://www.mdpi.com/1420-3049/29/24/5940/s1, Table S1: ^13^C NMR assignments of P(MMA-*co*-CEMA) (run 3); Table S2: ^13^C NMR assignments of P(MMA-*co*-MAOC) (run 6); Figure S1: GPC trace of run 1; Figure S2: GPC trace of run 2; Figure S3: GPC trace of run 3; Figure S4: GPC trace of run 4; Figure S5: GPC trace of run 5; Figure S6: GPC trace of run 6; Figure S7: TGA trace of run 1; Figure S8: TGA trace of run 2; Figure S9: TGA trace of run 3; Figure S10: TGA trace of run 4; Figure S11: TGA trace of run 5; Figure S12: TGA trace of run 6; Figure S13: Fluorescence micrographs of a pure PMMA sample; Figure S14: CEMA synthesis scheme; Figure S15: ^1^H NMR spectrum of CEMA; Figure S16: MAOC synthesis scheme; Figure S17: ^1^H NMR spectrum of MAOC; Figure S18: ^1^H NMR spectrum of run 3; Figure S19: ^1^H NMR spectrum of run 6. Figure S20: ^13^C NMR spectra of runs 1, 2, and 3 with integrals; Figure S21: ^13^C NMR spectra of runs 4, 5, and 6 with integrals.
