# Supplementary material for: Fluorescently Tagged Poly(methyl methacrylate)s
Source: Molecules. 2024 Dec 16;29(24):5940. doi: 10.3390/molecules29245940 (PMC11676142; doi:10.3390/molecules29245940)
Supplement: Supplementary file 1 [file molecules-29-05940-s001.zip › molecules-3328886-supplementary.pdf]

Supporting Information

Fluorescently Tagged Poly(methyl methacrylate)s

Fabia Grisi[1], Rubina Troiano[1], Donatella Fiore[2], Patrizia Gazzerro[2], Mariateresa Lettieri[3], Vincenzo Venditto[1] and Stefania Pragliola[1*]

[1] *Università di Salerno, Dipartimento di Chimica e Biologia, and INSTM Research Unit, Via Giovanni Paolo II 132, I-84084 Fisciano, SA, Italy.*
[2] *Università di Salerno, Dipartimento di Farmacia, Via Giovanni Paolo II, 132, I-84084 Fisciano, SA, Italy.*
[3] *CNR-SPIN, c/o Università di Salerno, Via Giovanni Paolo II, 1 32, I-84084 Fisciano, SA, Italy.*

Index

Table S1. ^{13}C NMR assignments of P(MMA-*co*-CEMA) (run **3**) p. 3
Table S2. ^{13}C NMR assignments of P(MMA-*co*-MAOC) (run **6**) p. 4
Figure S1. GPC trace of run **1** p. 5
Figure S2. GPC trace of run **2** p. 5
Figure S3. GPC trace of run **3** p. 5
Figure S4. GPC trace of run **4** p. 6
Figure S5. GPC trace of run **5** p. 6
Figure S6. GPC trace of run **6** p. 6
Figure S7. TGA trace of run **1** p. 7
Figure S8. TGA trace of run **2** p. 7
Figure S9. TGA trace of run **3** p. 7
Figure S10. TGA trace of run **4** p. 8
Figure S11. TGA trace of run **5** p. 8
Figure S12. TGA trace of run **6** p. 8
Figure S13. Fluorescence micrographs of a pure PMMA sample p. 9
Figure S14. CEMA synthesis scheme p. 10
Figure S15. ^{1}H NMR spectrum of CEMA p. 10
Figure S16. MAOC synthesis scheme p. 11
Figure S17. ^{1}H NMR spectrum of MAOC p. 11
Figure S18. ^{1}H NMR spectrum of run **3** p.12
Figure S19. ^{1}H NMR spectrum of run **6** p.12
Figure S20. ^{13}C NMR spectra of run **1**, **2**, and **3** with integrals p.13
Figure S21. ^{13}C NMR spectra of run **4**, **5**, and **6** with integrals p.14

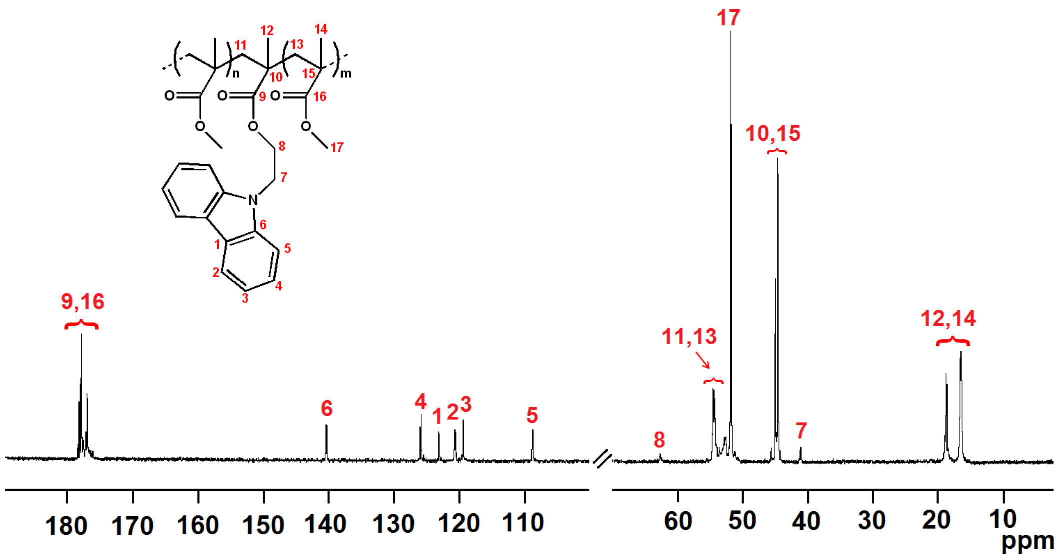

^{13}C NMR spectrum of run **3** ($CDCl_3$ solvent, TMS scale, 25 °C).

Table S1. ^{13}C NMR Assignments of Run 3

Carbon	Chem. Shift (ppm)
C1	122.960
C2	120.524
C3	116.353
C4	125.760
C5	108.718
C6	140.322
C7	40.935
C8	62.599
C9	178.207-176.104
C10	45.587-44.524
C11	54.492-52.631
C12	18.872-16.347
C13	54.492-52.631
C14	18.872-16.347
C15	45.587-44.524
C16	178.207-176.104
C17	51.568

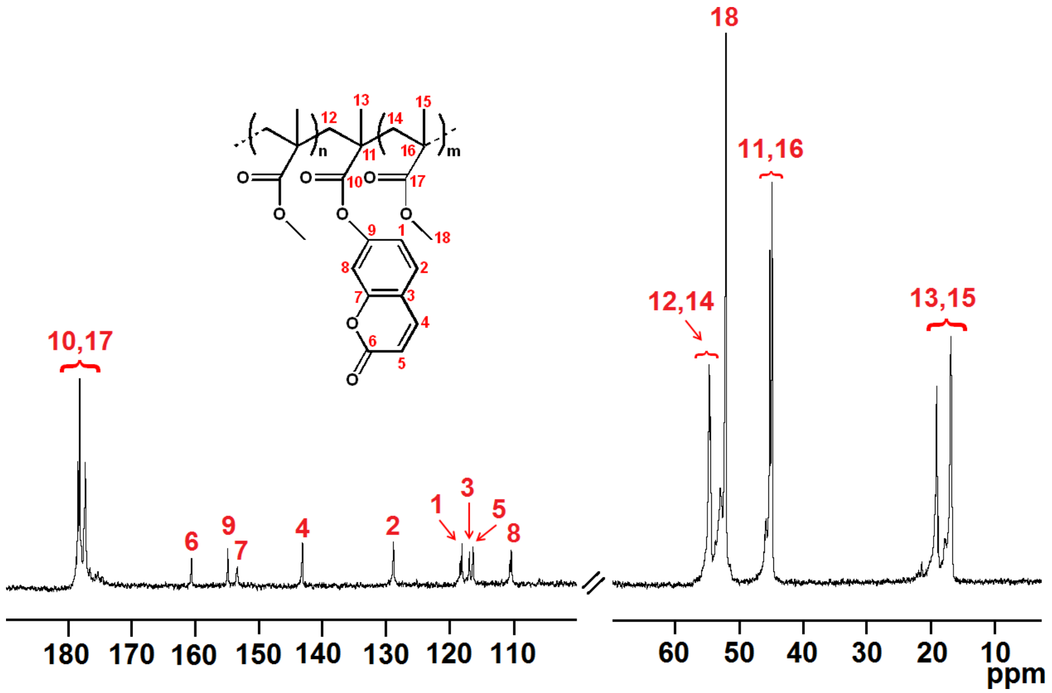

^{13}C NMR spectrum of run **6** ($CDCl_3$ solvent, TMS scale, 25 °C).

Table S2. ^{13}C NMR Assignments of Run 6.

Carbon	Chem. Shift (ppm)
**C1**	$\mathbf{117.80_{6}}$
**C2**	$\mathbf{128.57_{6}}$
**C3**	$\mathbf{116.65_{9}}$
**C4**	$\mathbf{142.81_{9}}$
**C5**	$\mathbf{116.12_{3}}$
**C6**	$\mathbf{160.21_{9}}$
**C7**	$\mathbf{153.13_{0}}$
**C8**	$\mathbf{110.06_{2}}$
**C9**	$\mathbf{154.57_{8}}$
**C10**	$\mathbf{178.34_{5}\text{-}176.94_{1}}$
**C11**	$\mathbf{45.48_{5}\text{-}44.52_{4}}$
**C12**	$\mathbf{54.39_{2}\text{-}52.63_{1}}$
**C13**	$\mathbf{18.87_{2}\text{-}16.34_{7}}$
**C14**	$\mathbf{54.39_{2}\text{-}52.63_{1}}$
**C15**	$\mathbf{18.87_{2}\text{-}16.34_{7}}$
**C16**	$\mathbf{45.48_{5}\text{-}44.52_{4}}$
**C17**	$\mathbf{178.34_{5}\text{-}176.94_{1}}$
**C18**	$\mathbf{51.83_{4}}$

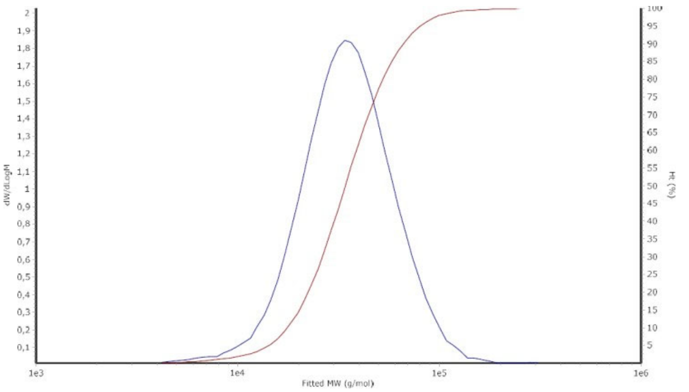

Figure S1. GPC trace of run **1**

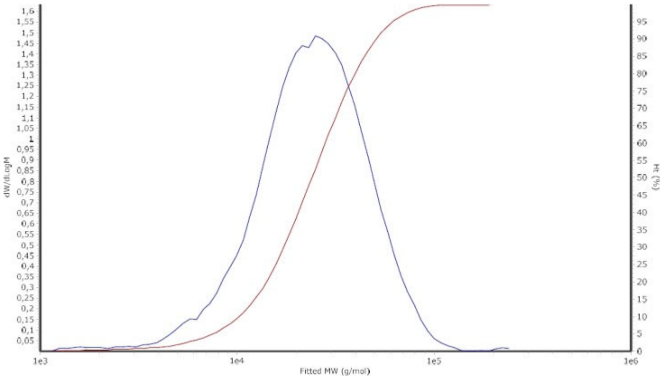

Figure S2. GPC trace of run **2**

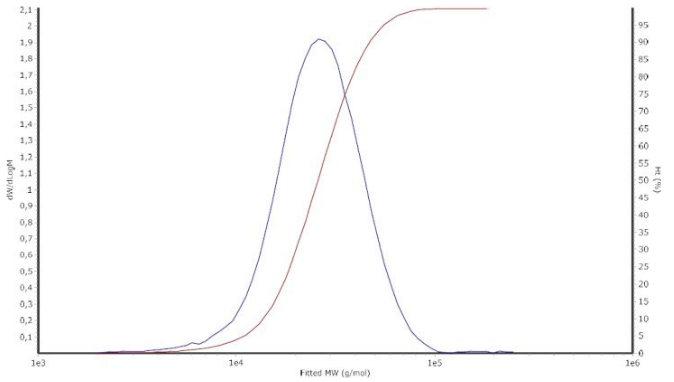

Figure S3. GPC trace of run **3**

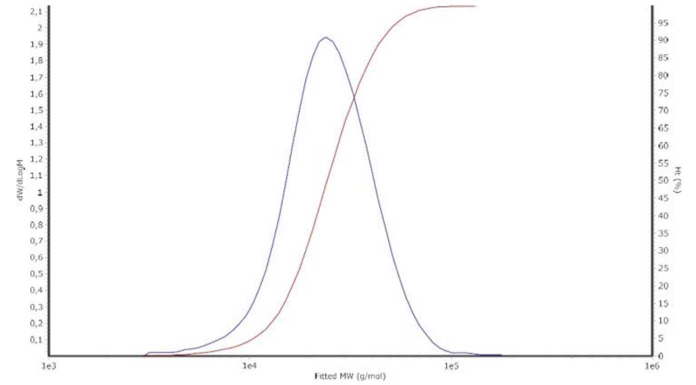

Figure S4. GPC trace of run **4**

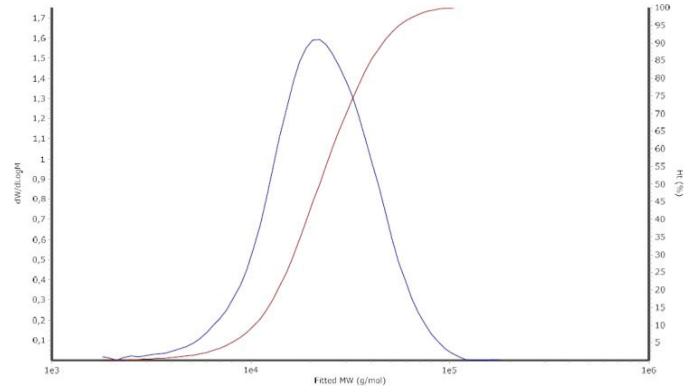

Figure S5. GPC trace of run **5**

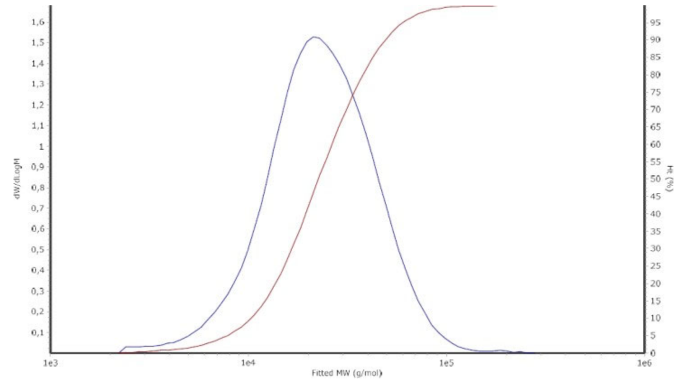

Figure S6. GPC trace of run **6**

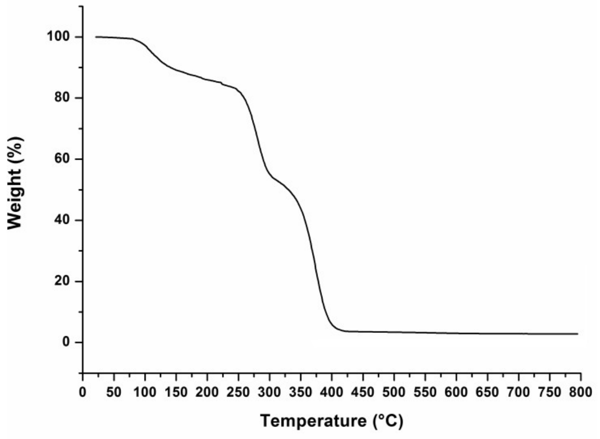

Figure S7. TGA trace of run **1**. (N_2, 10°C/min).

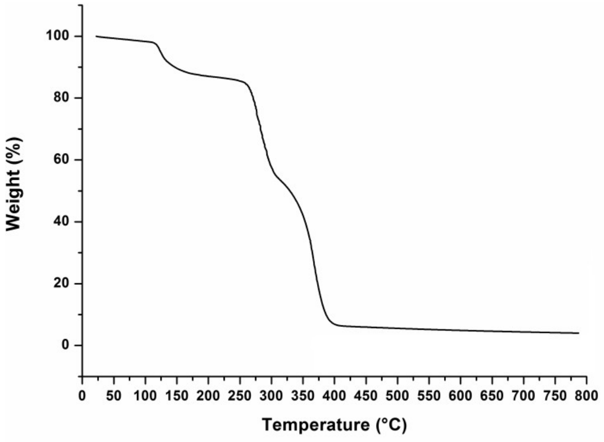

Figure S8. TGA trace of run **2**. (N_2, 10°C/min).

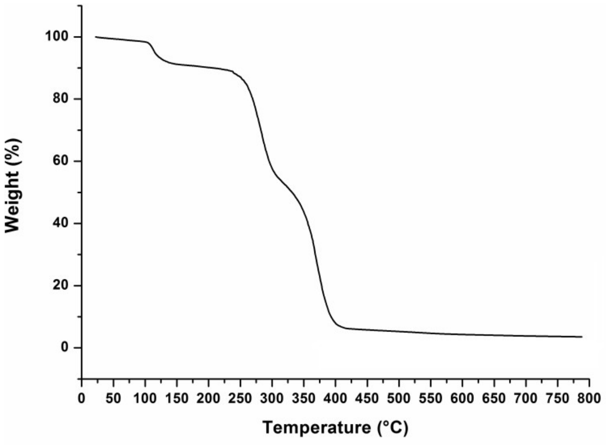

Figure S9. TGA trace of run **3**. (N_2, 10°C/min).

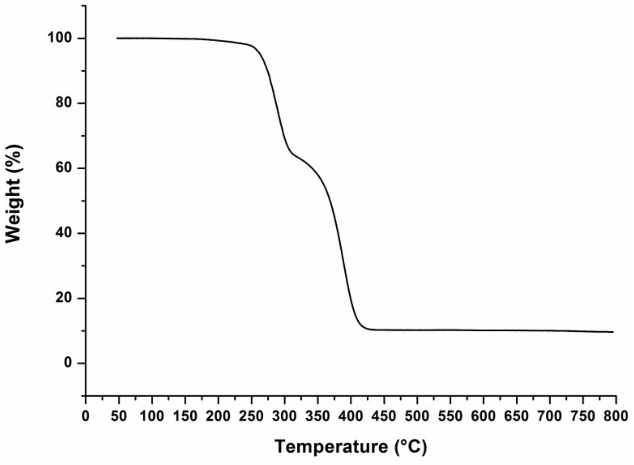

Figure S10. TGA trace of run **4**. (N_2, 10°C/min).

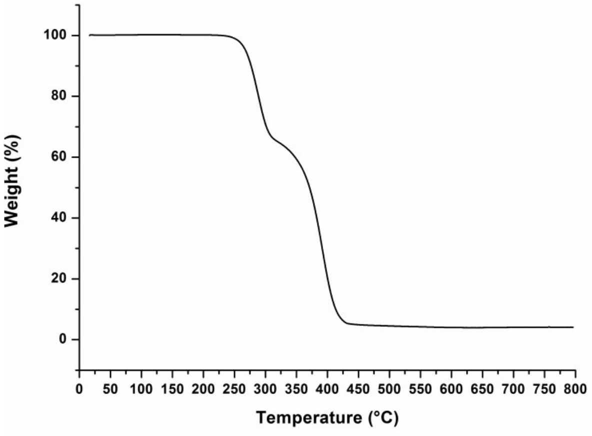

Figure S11. TGA trace of run **5**. (N_2, 10°C/min).

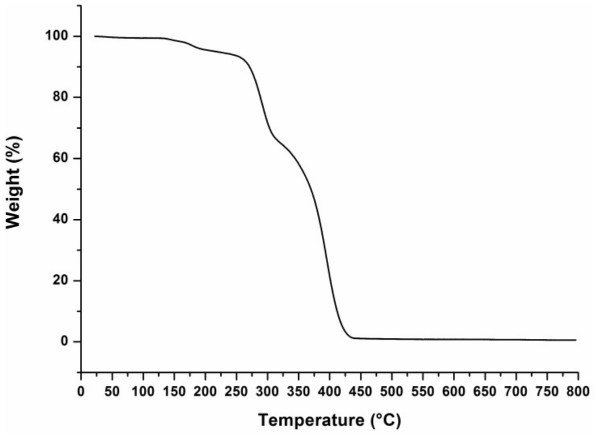

Figure S12. TGA trace of run **6**. (N_2, 10°C/min).

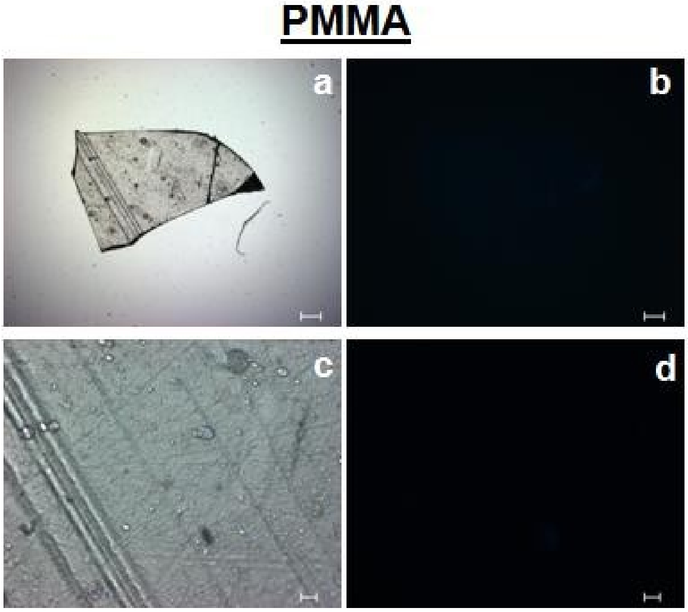

Figure S13. Representative images of negative control fluorescence properties. In a and c brightfield is shown. Panels b and d show blue fluorescence. Scale bars in a and b = 1000 µm; scale bars in c and d = 200 µm.

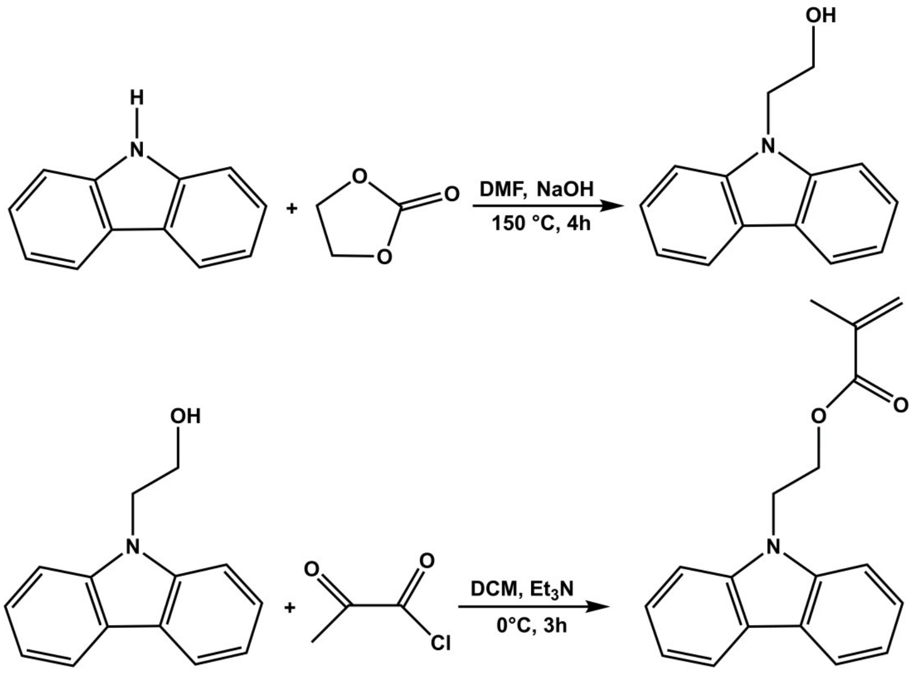

CEMA

Figure S14. CEMA synthesis scheme

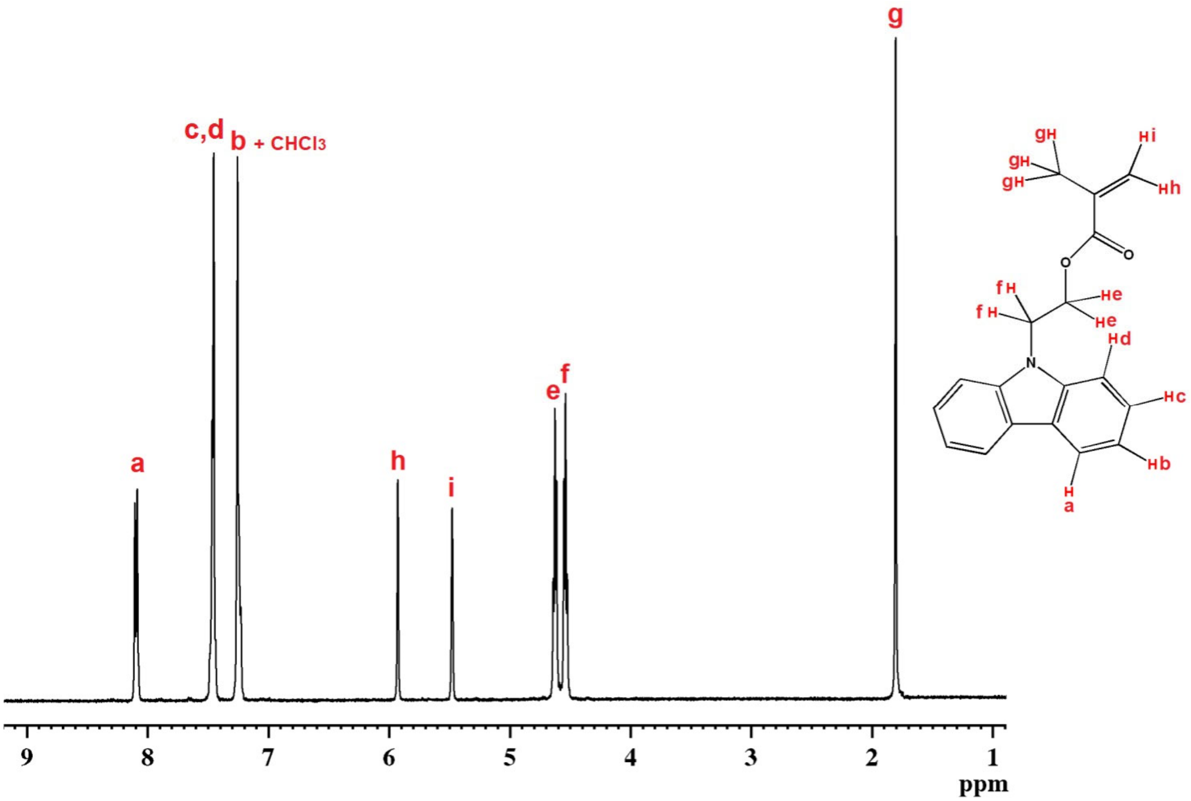

Figure S15. ^{1}H NMR spectrum of CEMA (solvent: $CHCl_3$, RT)

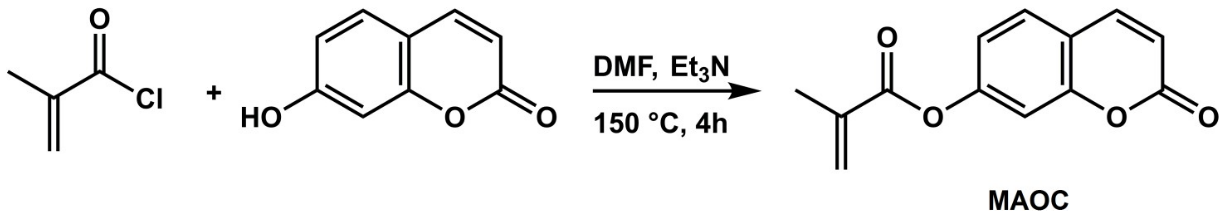

Figure S16. MAOC synthesis scheme

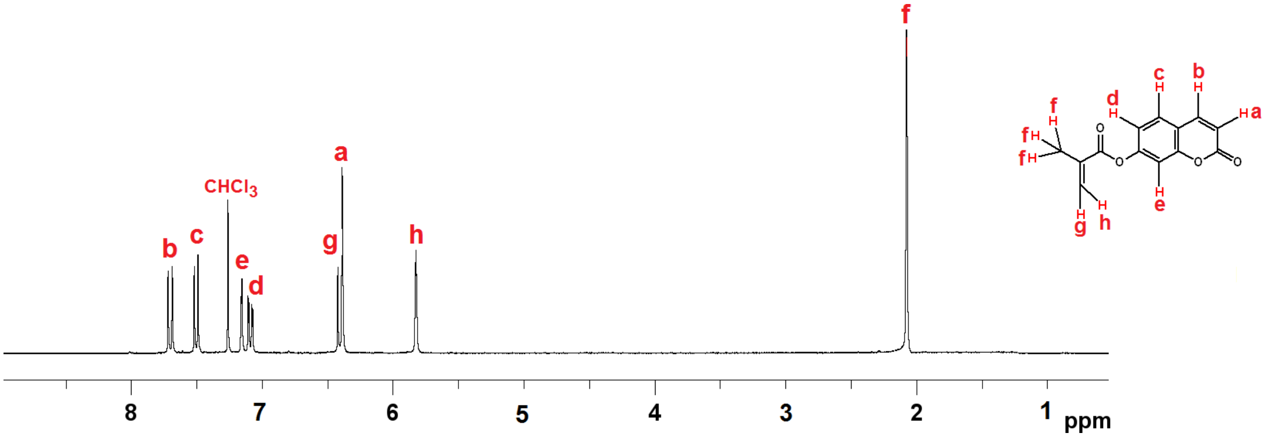

Figure S17. ^{1}H NMR spectrum of MAOC (solvent: $CHCl_3$, RT)

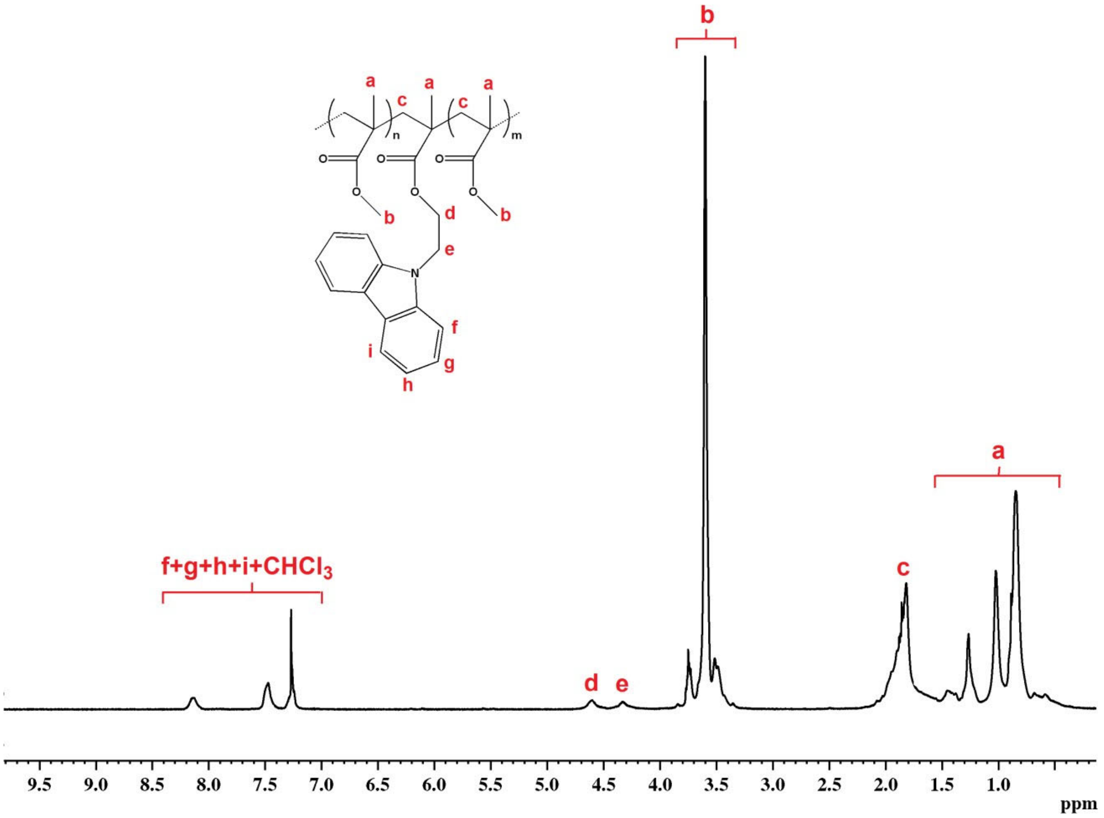

Figure S18. ^{1}H NMR spectrum of run **3** (solvent: $CHCl_3$, RT)

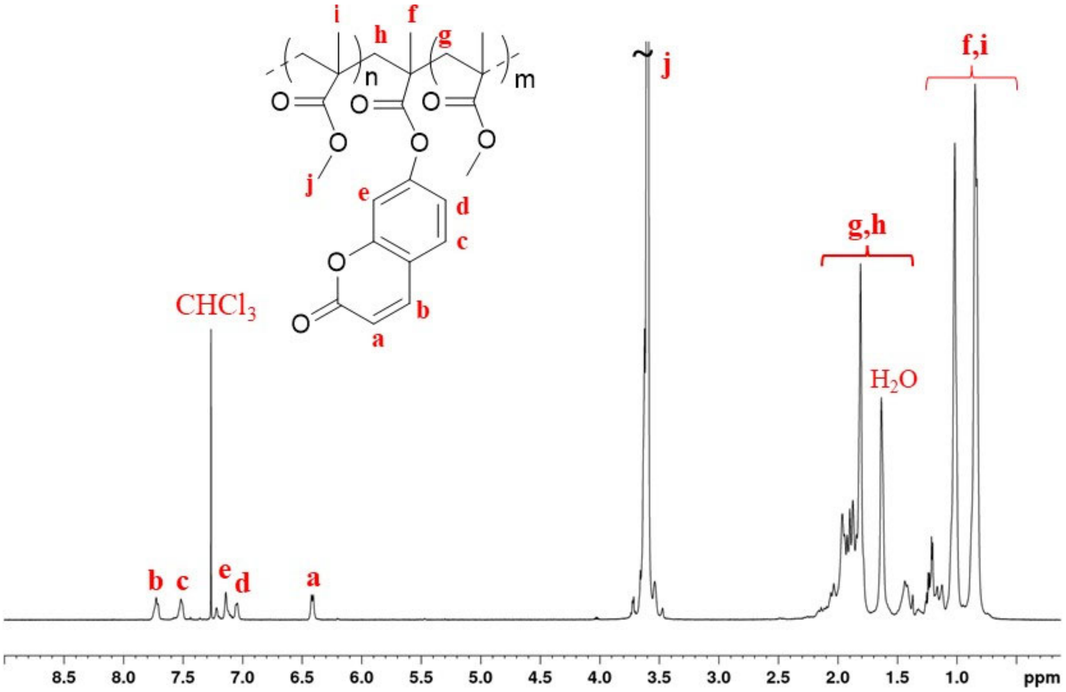

Figure S19. ^{1}H NMR spectrum of run **6** (solvent: $CHCl_3$, RT)

Run 3

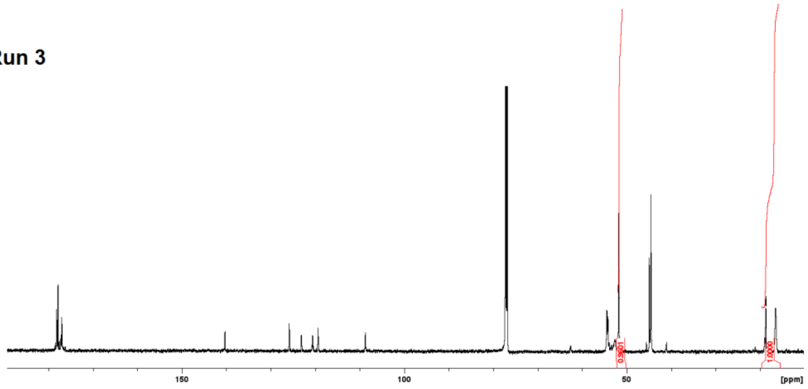

Run 2

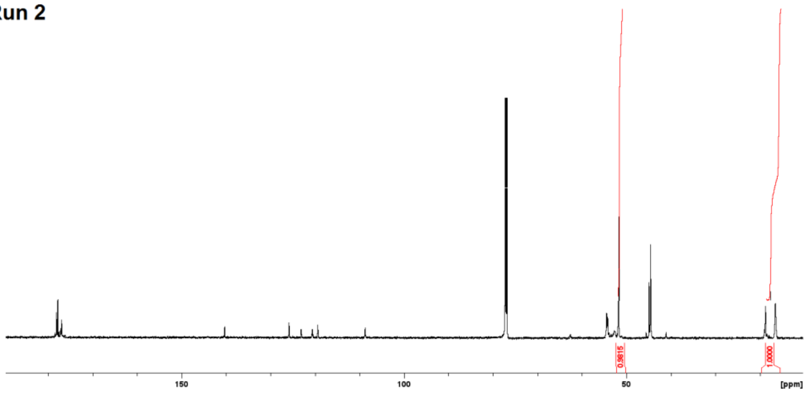

Run 1

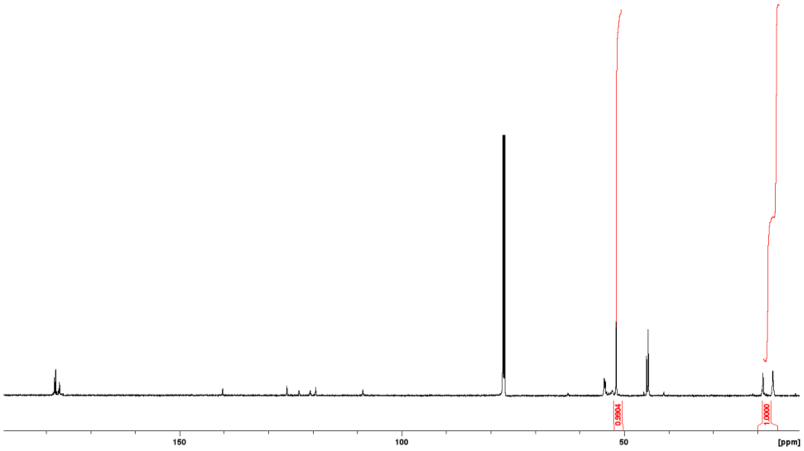

Figure S20. ^{13}C NMR spectra of run **1**, **2**, and **3** with integrals (solvent: $CHCl_3$, RT)

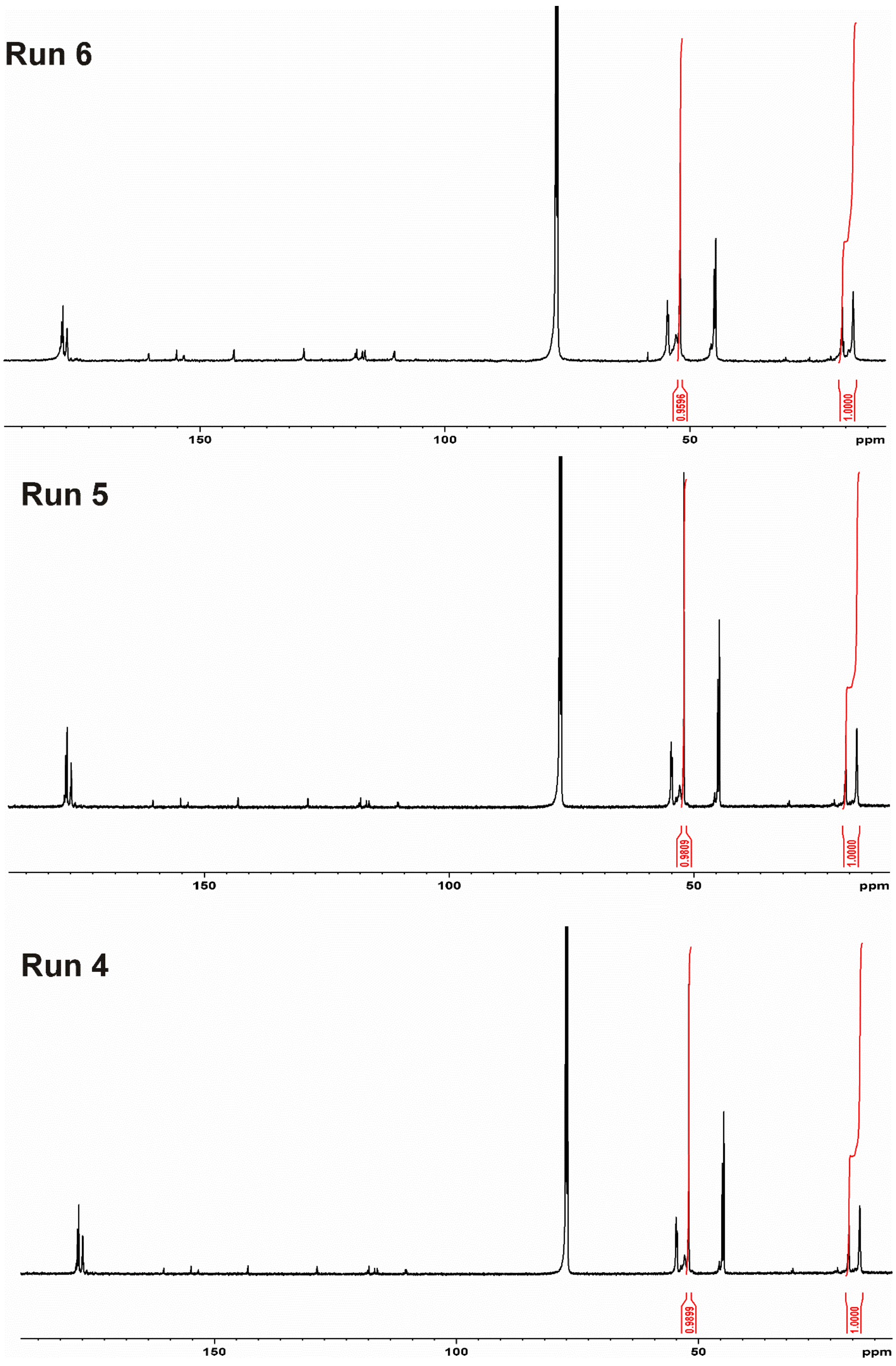

Figure S21. ^{13}C NMR spectra of run **4**, **5**, and **6** with integrals (solvent: $CHCl_3$, RT)
